# More active pre-school children have better motor competence at school starting age: an observational cohort study

**DOI:** 10.1186/s12889-016-3742-1

**Published:** 2016-10-10

**Authors:** Lisa M. Barnett, Jo Salmon, Kylie D. Hesketh

**Affiliations:** 1 School of Health and Social Development, Deakin University, 221 Burwood Hwy, Burwood, VIC Australia; 2 Institute for Physical Activity and Nutrition, School of Exercise and Nutrition Sciences, Deakin University, 221 Burwood Hwy, Burwood, VIC Australia

**Keywords:** Physical activity, Object control skill, Longitudinal, fundamental movement skill, Early childhood, Toddlers

## Abstract

**Background:**

Almost half of young children do not achieve minimum recommendations of 60 daily minutes in physical activity. Physical activity is potentially an important determinant of the development of motor competence in children. This study is one of very few longitudinal studies in this area and the first to investigate early childhood physical activity as a predictor of subsequent motor skill competence.

**Methods:**

Children were assessed as part of the Melbourne InFANT Program longitudinal cohort study at 19 months, 3.5 years and 5 years. Moderate-to-vigorous physical activity (MVPA) (accelerometry) was assessed at each time point. At age 5, children were also assessed in actual (Test of Gross Motor Development-2) and perceived motor competence (Pictorial Scale of Perceived Movement Skill Competence). General linear models were performed with all 12 skills (six object control and six locomotor skills), both actual and perceived, at age 5 as the respective outcome variables. Predictor variables alternated between MVPA at 19 months, 3.5 years and 5 years.

**Results:**

Based on standardized TGMD-2 scores most children were average or below in their skill level at age 5. MVPA at 19 months was not a predictor of actual or perceived skill at age 5. MVPA at 3.5 years was associated with actual locomotor skill (B = 0.073, *p* = 0.033) and perceived total skill at 5 years of age (B = 0.059, *p* = 0.044). MVPA was not a predictor of actual or perceived object control skill at any age.

**Conclusion:**

Parents and preschool staff should be informed that more time in MVPA as a preschool child contributes to locomotor skill and to perceptions of skill ability in a child of school starting age. Understanding this relationship will assist in intervention development.

## Background

Just under half of children aged from 2 to 6 years achieve 60 min per day in physical activity [1]. Given that this is a minimum recommendation in this age range, this is a serious concern. Focusing on physical activity promotion in this age range is important as physical activity behaviors established during early childhood may track into later childhood [2] and adolescence [3].

Motor competence in children has gained credence in the last decade as an important correlate of physical activity and other health related behaviours and outcomes [4], including higher cardiorespiratory fitness and healthier weight status [5]. Motor competence is a broad term that encompasses fundamental movement skill ability, including locomotor, object control and stability factors [6], and motor coordination [4].

Previously it has been hypothesized that children with better motor competence participate in higher levels of physical activity and that this in turn helps to further develop higher actual and perceived motor competence [7]. Countless sporting activities and games need competence in fundamental motor skills (e.g., running, jumping, catching, throwing) to enable physical activity participation [4]. Now, close to a decade later, there is convincing evidence that physical activity and actual motor skill competence are associated in children and youth [4, 5, 8]. In addition, high perceptions of physical competence contribute to increased physical activity in children and youth [9]. Yet as most evidence is cross-sectional, the relationship between physical activity and actual and perceived motor competence is not fully understood.

The inherent hypothesis posed by Stodden and colleagues [7] in terms of the relationship between motor competence and physical activity being important in both directions has had limited testing [4, 10]. More is known about the skill to physical activity relationship than the physical activity to skill relationship. These relationships are hypothesized to differ at different stages, i.e., engagement in physical activity is important for the development of motor competence but then as children develop, motor competence is of more importance for physical activity participation. In a path analysis study in adolescents, Barnett and colleagues found a reciprocal relationship between object control skill competence and physical activity, but a one way relationship from physical activity to locomotor skills. Perceived competence acted as a mediator in both proposed path directions, although the strength differed [10].

There are few longitudinal studies to draw from. The northern Finland Birth Cohort found that age at walking supported and age at standing unaided predicted sports participation at 14 years [11]. Studies in older children found childhood motor skill competence (age 10 and age six respectively) was a predictor of subsequent physical activity [12, 13] and physical activity in Grade 7 was a predictor of motor competence in Grade 8, but only for boys [14]. No longitudinal study to our knowledge has examined physical activity as a determinant of actual or perceived motor competence in very young children. A systematic review in preschool children determined physical activity was a key cross-sectional correlate of motor competence [15] indicating that it is worthwhile to investigate such associations at this young age. Understanding more about the role of physical activity in the development of motor competence in young children will potentially help inform recommendations and advice to parents, carers, health professionals and educators of children in this age group. This study therefore sought to investigate whether physical activity in the toddler and/or preschool years influences subsequent motor skill competence at school starting age (5 years). The hypothesis was that physical activity behaviour would predict actual and perceived motor competence.

## Methods

### Participants and setting

Children were part of the Melbourne Infant Feeding, Activity and Nutrition Trial (InFANT). The Melbourne InFANT Program was originally a 15-month obesity prevention intervention targeted to first-time parents and their infants from 4 months of age from 2008 to 2010, with post-intervention follow up at child age 19 months, 3.5 years and 5 years [16]. Parents were recruited through first time parent groups operated by universal access child health centers in randomly selected geographically based government areas. The intervention was conducted with 542 parent–child pairs from 62 different parent groups, and at intervention conclusion there were a total of 492 families still enrolled. Data for these analyses are drawn from three follow-up time points: 19 months, 3.5 years and 5 years of age. Children from both intervention and control groups were included as for the current study it is not important to distinguish between these groups, although all models did adjust for intervention status. Children completed physical activity assessment at 19 months, 3.5 and 5 years. Children completed actual and perceived motor competence assessments at 5 years only in 2013.

### Measures

Parents completed a written self-report questionnaire, which provided demographic information about the responding parent (parent country of birth, language spoken most at home, residential socioeconomic position, employment when child was 5 years old, and highest level of education) [16]. At 19 months, 3.5 and 5 years children’s physical activity levels were measured every 15 s for eight consecutive days using hip-mounted ActiGraph GT1M accelerometers. Children were instructed to wear the monitor during all waking hours except during water-based activities. The total time spent in moderate-vigorous intensity physical activity per day (MVPA) was the predictor variable in each model. The average time spent in MVPA per day and average daily wear time were computed using data collected on each valid day (defined as 444+ mins, 7.4 h of data). To be included in analyses, children were required to have worn the monitor for any four days or more; this could include weekend or weekdays.

Accelerometer data were initially downloaded using ActiLife Software (version 5.10) and then analyzed using customised Excel macros. At each assessment point, non-wear time was defined as 20 min of consecutive zeroes; commonly used to define non-wear in children [17]. The Evenson cut points were used to define MVPA (2296–4011 counts per minute [cpm] for moderate, and ≥4012 cpm for vigorous) for all three time points [18]. These cut-points have been validated and found to provide acceptable classifications of MVPA in toddlers [19] and children [20].

At age 5, children were assessed using the Test of Gross Motor Development- 2nd Edition (TGMD-2) on 12 skills; six locomotor (run, gallop, hop, leap, horizontal jump, and slide) and six object control skills (striking a stationary ball, stationary dribble, kick, catch, overhand throw, and underhand roll) according to established protocols [21]. Each skill was performed twice and each attempt was scored with each component of the skill receiving a ‘1’ if correctly executed or a ‘0’ if not. Scores of the two trials were summed to obtain a raw score for each skill. Each actual skill score was summed (total skill possible range 0–96, locomotor 0–48, object control 0–48). Object control and locomotor scores were also standardized by age and sex according to the TGMD-2 protocol [21] for descriptive information only. All children were assessed in the home setting and assessments were video recorded. Videos were later coded by two experienced raters who had been shown to have acceptable intra-rater reliabity in other studies [22]. Inter-rater reliability using Intra Class Correlations (ICC) was assessed on a subsample of 30 children in this study at age 5 years for all 12 skills (ICC = 0.76, 95 % CI 0.56, 0.88).

At age 5, the Pictorial Scale for Perceived Movement Skill Competence for Young Children (PMSC) was used to assess children’s perceptions of their own motor competence. This pictorial instrument assesses the same 12 skill items as the TGMD-2. Skills for each subscale on the PMSC are ordered so that a cartoon image of a child performing a skill competently is next to an image of a child performing a skill not as competently [23]. First, children select between the picture portraying a child who is competent in a skill and the picture portraying a child who is not so competent. Whilst the child is looking at the selected picture they are then asked to discriminate their level of competence: “really good” versus “pretty good”, or “not that good” versus “sort of good”, dependent on their initial competence response. For example, “this child is pretty good at hopping, this child is not that good at hopping, which child is like you?”. If children select the competent picture they are asked: ‘are you really good at…’ (score of four) or ‘pretty good at….’ (score of three), if children pick the not so competent picture, they are asked: ‘are you not that good at (score of one) or sort of good at….’ (score of two). The result is a four point Likert scale variable (range 1–4). Whilst the score for each item cannot be considered as continuous, the perceived skill scores for each item are then summed to provide a total score (possible range 12–48), locomotor score (6–24) and object control score (6–24); and these scores are treated as continuous. An extensive description of the PMSC protocol is provided elsewhere [24]. Previous research has established acceptable test-retest reliability, internal reliability and face validity of the 12 skills items in the PMSC [24]. Construct validity for the perceived skills has also been found to be acceptable using the data from the current study [25].

### Analysis

Of the 492 families enrolled at intervention completion (age 19 months), over half this group (*n* = 272, 55.3 %) were assessed at 5 years of age for actual and perceived competence. To be included in the current analysis children also needed to have data for MVPA at *any of* the respective predictor time points (19 months, 3.5 years and 5 years). One outlier was excluded from the 19 month old models due to uncharacteristically high physical activity (considered likely to be spurious). Another outlier was excluded due to an extremely uncharacteristic low perceived skill score. As the summed perceived skill score was negatively skewed, models were run with the untransformed variable and the log transformed variable; if there was no difference in significance and estimates, the model with the untransformed variable was presented for ease of interpretation. Independent t tests were used to check whether a) children with valid physical activity data at 19 months, 3.5 years and 5 years were any different in terms of actual movement skill at age 5 years to those children without valid physical activity data and b) more active children remained in the study at age 5 years (using MVPA at first measurement i.e., 19 months).

Initially linear mixed models were performed which enabled variance at the level of parent group to be accounted for. Models had a) the sum of 12 skills, locomotor and object control at age 5 (using the raw scores) and b) the sum of 12 perceived skills, locomotor and object control as the outcome variables. Model 1 had MVPA at 19 months as the predictor (*n* = 185), Model 2 had MVPA at 3.5 years (*n* = 116) and Model 3 had MVPA at age 5 years (*n* = 126). Each model adjusted for original intervention/control classification, sex, age and valid monitor wear time days. The models with MVPA at 19 months as the predictor also adjusted for age at first walking, a potential confounder of infant MVPA [26]. When parent group was found to not account for any variance, models were all rerun as general linear models. The parameter estimates did not differ between the simple and mixed models, but the standard errors were inflated in the mixed models, hence a simple fixed effects model was preferred.

## Results

There were no differences in actual movement skill at age 5 between children who had valid physical activity data and children who did not at any time point. Those who remained in the sample had a trend to be less active at 19 months old than those who dropped out (28 min per day compared to 25 min, *p* = 0.050).

Descriptive data for the parent sample (based on the time point with the largest number of parents) is presented in Table 1. Parent respondents were largely mothers born in Australia who spoke English at home. Around 60 % of respondent parents were working part- or full-time and a similar proportion had a university education.

**Table 1:** Descriptive data for the parent sample that were included when their child was 19 months (*n* = 193)

Characteristic		n	%
Residential Socioeconomic position	High	37	19.2
	Medium	123	63.7
	Low	33	17.1
Respondent Parent	Mother	184	95.3
Country of birth	Australia	158	81.9
Main language spoken at home	English	186	96.4
Employment status	Working full or part time	113	58.5
Education level	University degree or higher	116	60.1

Most children (*n* = 164, 84.0 %) were not attending school at 5 years old. Children’s MVPA at each time point and actual and perceived skills at age 5 are described in Table 2. Children were least active at 19 months, and most active at 5 years old. Average perceived skill was 41.4 out of a possible 48 (range of 29 to 48), showing children tended to respond on average between ‘pretty good’ and ‘really good’ for each skill. Based on standardized TGMD scores nearly all children were average (object control *n* = 81, 63.8 %, locomotor *n* = 87, 68.5 %), or below average (object control *n* = 35, 27.6 %, locomotor *n* = 31, 24.4 %) in their skill level at age 5.

**Table 2:** Child sample, MVPA at each time point and actual and perceived skills at age 5

Sample					MVPA	Actual skill			Perceived skill		
Age	Total	Boys	Girls	Proportion of InFANT sample (*n* = 492)	Minutes p/day	Total (0–96)	Locomotor (0–48)	Object Control (0–48)	Total (12–48)	Locomotor (6–24)	Object Control (6–24)
M (SD)	N	n (%)	n (%)	%	M (SD)	M (SD)	M (SD)	M (SD)	M (SD)	M (SD)	M (SD)
19.0 mths (2.2)	193	98 (50.8)	95 (49.2)	39.2	25.1 (9.4)	–	–	–	–	–	–
3.5 years (0.2)	118	53 (44.9)	65 (55.1)	24.0	42.5 (16.1)	–	–	–	–	–	–
5.0 years (0.1)	127	59 (46.5)	68 (53.5)	25.8	52.8 (17.9)	49.7 (9.7)	26.0 (5.5)	23.3 (6.1)	41.4 (4.7)	21.1 (2.3)	20.3 (2.9)

### MVPA as a predictor of total skill competence at age 5 years

MVPA at 19 months was not a predictor of total skill (actual or perceived) at age 5. MVPA at 3.5 years was predictive of perceived total skill at age 5 years (B = 0.059, *p* = 0.044) and approached significance for actual skill at age 5 years (B = 0.109, *p* = 0.059). MVPA at 5 years was not associated with total skill (actual or perceived). See Table 3.

**Table 3:** MVPA at each age (19 months, 3.5 years, 5 years) as a predictor of actual total skill and perceived total skill at age 5 years

MVPA 19 mths as predictor of: (corrected total *n* = 185)				MVPA 3.5 years as predictor of: (corrected total *n* = 116)				MVPA 5 years as predictor of: (corrected total *n* = 126)			
B	Std error	P	95 % LCI, UCI	B	Std error	P	95 % LCI, UCI	B	Std error	P	95 % LCI, UCI
Actual Total Skill Age 5											
−.116	.073	.117	−.260,.029	.109	.057	.059	−.004,.222	.078	.050	.121	−.021,.178
Sex (boy)*, Age*.				No sig. adjustment variables				No sig. adjustment variables			
Perceived Total Skill Age 5											
−.028	.037	.446	−.102,.045	.059	.029	.044*	.002,.116	.016	.025	.514	−.033,.066
No sig. adjustment variables				No sig. adjustment variables				No sig. adjustment variables			

Note. *LCI* lower confidence interval, *UCI* upper confidence interval. All models adjusted for monitor wear time and age at the time MVPA was assessed, sex of child and original treatment group. The models at 19 months old also adjusted for age at first walking. Significant adjustment variables are specified with *p < 0.05

Any model with perceived competence as the outcome in which MVPA was significant or close to significance (*p* < .1) was rerun with the transformed log of perceived competence. As the outcome did not change, the untransformed variable is used for ease of interpretation

### MVPA as a predictor of object control skill at age 5 years

MVPA at any age was not associated with object control skill (actual or perceived) at age 5 years. See Table 4.

**Table 4:** MVPA at each age (19 months, 3.5 years, 5 years) as a predictor of actual and perceived object control skill at age 5 years

MVPA 19 mths as predictor of: (corrected total *n* = 185)				MVPA 3.5 years as predictor of: (corrected total *n* = 116)				MVPA 5 years as predictor of: (corrected total *n* = 126)			
B	Std error	P	95 % LCI, UCI	B	Std error	P	95 % LCI, UCI	B	Std error	P	95 % LCI, UCI
Actual Object Control Skill Age 5											
−.061	.046	.185	−.153, .030	.032	.034	.348	−.035,.098	.029	.031	.356	−.033,.090
Age*, Sex (Boy)**				Age*, Sex (Boy)*				Sex (Boy)*			
Perceived Object Control Skill Age 5											
−.032	.022	.151	−.077,.012	.033	.017	.061	−.002,.067	.011	.015	.483	−.020, .041
Sex (Boy)*				Treatment group (Control)*				No sig. adjustment variables			

Note. *LCI* lower confidence interval, *UCI* upper confidence interval. All models adjusted for monitor wear time and age at the time MVPA was assessed, sex of child and original treatment group. The models at 19 months old also adjusted for age at first walking. * *p* < 0.05, **p < 0.001

Any model with perceived competence as the outcome in which MVPA was significant or close to significance (*p* < .1) was rerun with the transformed log of perceived competence. As the outcome did not change the untransformed variable is used for ease of interpretation

### MVPA as a predictor of locomotor skill at age 5 years

MVPA at 19 months was not a predictor of locomotor skill (actual or perceived) at age 5. MVPA at 3.5 years was predictive of actual locomotor skill at age 5 years (B = 0.073, *p* = 0.033), but not perceived locomotor skill. At 5 years, MVPA was not cross-sectionally associated with locomotor skill (actual or perceived). See Table 5.

**Table 5:** MVPA at each age (19 months, 3.5 years, 5 years) as a predictor of actual and perceived locomotor skill at age 5 years

MVPA 19 mths as predictor of: (corrected total *n* = 185)				MVPA 3.5 years as predictor of: (corrected total *n* = 116)				MVPA 5 years as predictor of: (corrected total *n* = 126)			
B	Std error	P	95 % LCI, UCI	B	Std error	P	95 % LCI, UCI	B	Std error	P	95 % LCI, UCI
Actual Locomotor Skill Age 5											
−.048	.042	.251	−.131, .035	.073	.034	.033*	.006,.139	.043	.029	.134	−.014,.100
No sig. adjustment variables				Sex (Girl)*				No sig. adjustment variables			
Perceived Locomotor Skill Age 5											
.004	.019	.842	−.034,.042	.026	.015	.085	−.004,.056	.006	.012	.651	−.019, .030
No sig. adjustment variables				No sig. adjustment variables				No sig. adjustment variables			

Note. *LCI* lower confidence interval, *UCI* upper confidence interval. All models adjusted for monitor wear time and age at the time MVPA was assessed, sex of child and original treatment group. The models at 19 months old also adjusted for age at first walking. Significant adjustment variables are specified with *p < 0.05

Any model with perceived competence as the outcome in which MVPA was significant or close to significance (*p* < .1) was rerun with the transformed log of perceived competence. As the outcome did not change the untransformed variable is used for ease of interpretation

## Discussion

This study explored whether early physical activity behaviour impacts on subsequent actual or perceived motor competence. We found that children had positive perceptions overall. MVPA at age 3.5 years was predictive of perceived total skill. MVPA in preschool years predicted locomotor but not object control skills. Stodden et al. [7] postulated that, in young children, engagement in physical activity is important for the development of motor competence but then as children develop, motor competence is of more importance for physical activity participation. Our findings support the first part of this hypothesis, in that MVPA in the preschool years did contribute to subsequent motor competence in the current sample. A recent review concluded that whilst there is strong evidence of an association between motor competence and physical activity (e.g., [5, 8, 27]), there were still questions in terms of antecedent/consequent mechanisms [4]. Based on our sample, a preschool child who spent 15 min a day more in MVPA would demonstrate approximately one unit higher for locomotor competence. This is similar to performing one additional component correctly in one of the six locomotor skills (e.g., “both feet come off the floor together and land together” in the jump). This may be meaningful, as mastering particular components may enhance play and game opportunities. For instance being able to do a ‘step hop’ sequence means being able to play games involving the skip. As such, this finding warrants recommending to parents and preschool staff that MVPA at this age does make a difference to skill outcomes.

Other research has demonstrated ‘free play’ does not contribute much to motor skill competence [28], so it is suggested that in very young children the type and quality of physical activity relate more to motor skill development than simply movement quantity and intensity. This may explain why the relationship found in this study was with subsequent locomotor competence rather than object control competence. This would be expected as the activities that preschool-aged children are likely to engage in are more locomotive in nature (e.g., running and playing in the garden). High participation in ball games would be more likely to be associated with better object control competence. One study investigated different types of organized physical activity participation in preschool aged children (dance, ‘kindy gym’, swimming) and found different associations in terms of object control and locomotor competence [29]. This provides further evidence that young children need specific opportunities for skills to be taught and practised. Whilst objective measurement of physical activity has many advantages, it does not enable isolation of the types and quality of physical activity that children are engaging in. A recent systematic review of motor skill correlates in children and youth found that whilst physical activity was a correlate of some aspects of motor competence (i.e., total skills and motor coordination), evidence was indeterminate for physical activity being a correlate of object control or locomotor skill competence [30]. This illustrates further that when examining relationships between physical activity and motor competence, that it is important to examine relationships according to the way motor competence is operationalized.

Stodden and colleague’s model [7] also hypothesized that relationships between motor competence and physical activity increases in strength as children age and develop. A recent narrative review supports this premise, with the conclusion that overall, whilst data strongly supports a positive relationship between motor competence and physical activity, associations are low to moderate in early to middle childhood [4]. At first glance our null findings at age 5 years appears counter intuitive, and contrary to other investigations (e.g., [29, 31]) but it could also be viewed that previous time in MVPA is more important to the current level of skill than any pattern of current MVPA. Motor competence needs time to develop and if we also consider that MVPA only tracks to a moderate level in early childhood [32], it is reasonable that previous time in MVPA may be more important in some cases to skill development than current time in MVPA.

It is likely that the null finding in terms of toddler activity being a predictor of subsequent motor competence is heavily influenced by the developmental stage of children at this early age. Even though we adjusted for age at first walking, many children would still be using crawling as a common form of locomotion and that behaviour is not picked up well by accelerometers. Since children’s activity levels are so influenced by their developmental stage at this age they therefore may not be a true reflection of characteristic physical activity levels of each individual child. This may help to explain why we found no relationships between toddler MVPA and subsequent actual and perceived skill.

Few studies have investigated perceived physical competence and physical activity in preschool age children [33]. Those studies which have specifically examined perceived motor competence (rather than general perceptions of physical competence), have focused on an older group of children (first three years of school) [34, 35]. Cross-sectional findings have suggested that whilst school children’s perceptions of their motor competence are associated with their actual skills [34, 35], perceptions did not relate to their current physical activity levels [34, 36]. Yet, in the present sample, MVPA among preschool-aged children was associated with subsequent overall perceived motor competence at age 5 years. Our finding can be interpreted as a child who spends 15 more minutes in MVPA per day would be approximately one unit higher in perceived competence; similar to shifting from a self-perception of ‘sort of good’ to ‘pretty good’ in one of the 12 skills. This confirms what was found with actual skill, in that previous physical activity behaviour appears (in this sample) to have more influence on children’s perceptions than current physical activity behaviour. Considering that developmentally children tend to rate themselves favourably, this indicates that the amount of time in MVPA still has the ability to discriminate between children with different self-perceptions. This finding is meaningful, as a child who believes they are ‘pretty good’ at catching as opposed to ‘sort of good’, may potentially be more motivated to participate in a whole range of ball sports that involve catching.

The strengths of the current study include objective measurement of physical activity at three time points in a good sized sample which reflects well on generalizability of findings. A further strength is the use of an instrument to assess perceived motor competence aligned with a measure of actual motor competence. Limitations are that actual and perceived motor competence were only measured at age 5 years. This meant a mixed model which investigated change over time could not be used. Whilst there are instruments which could have assessed motor competence broadly at each time point, it was not possible for fundamental movement skill competence to be assessed at each time point as the TGMD-2 is only suitable to a minimum age 3 years, and motor milestones are relevant at age 19 months. The TGMD-2 is the only instrument to our knowledge which comprehensively assesses a broad range of fundamental movement skills in young children. It was not possible to measure perceived motor competence prior to age 5 as younger children could not be expected to complete this assessment due to their cognitive developmental stage. The number of children with valid physical activity assessments at each time point (43–71 % of age 5 sample) is a limitation, however there were no differences in skill between those with valid assessments and those without, showing this did not likely influence internal validity of results. Although those who stayed in the study were less physically active at 19 months old.

## Conclusions

This study has added to the literature by demonstrating for the first time that in preschool aged children the amount of time in MVPA is important to subsequent actual and perceived motor competence. No other longitudinal study to the authors’ knowledge has investigated physical activity as a predictor of actual or perceived motor competence in young children. Based on this study it is important to recommend to parents and preschool/child care staff that children should be encouraged to spend as much time as possible in MVPA. It is acknowledged that for young children this will likely be achieved through short sporadic bursts of activity. Future research may seek to investigate, what *type* of physical activity is of most importance to subsequent skill development.

## Abbreviations

ICC: Intra Class Correlations
InFANT: Melbourne Infant Feeding, Activity and Nutrition Trial
MVPA: Moderate-to-vigorous physical activity
PMSC: Perceived Movement Skill Competence for Young Children
TGMD-2: Test of Gross Motor Development- 2nd Edition

## References

[1] Tucker P (2008). The physical activity levels of preschool-aged children: a systematic review. Early Child Res Q.

[2] Janz KF, Burns TL, Levy SM (2005). Tracking of activity and sedentary behaviors in childhood: The Iowa Bone Development study. Am J Prev Med.

[3] Kristensen PL, Moller NC, Korsholm L, Wedderkopp N, Andersen LB, Froberg K (2008). Tracking of objectively measured physical activity from childhood to adolescence: the European youth heart study. Scand J Med Sci Sports.

[4] Robinson LE, Stodden DF, Barnett LM, Lopes VP, Logan SW, Rodrigues LP, D’Hondt E (2015). Motor competence and its effect on positive developmental trajectories of health. Sports Med.

[5] Lubans DR, Morgan PJ, Cliff DP, Barnett LM, Okely AD (2010). Review of the benefits associated with fundamental movement skill competency in youth. Sports Med.

[6] Gabbard C (2011). Lifelong motor development.

[7] Stodden DF, Goodway JD, Langendorfer SJ, Roberton MA, Rudisall ME, Garcia C, Garcia LE (2008). A developmental perspective on the role of motor skill competence in physical activity: an emergent relationship. Quest.

[8] Holfelder B, Schott N (2014). Relationship of fundamental movement skills and physical activity in children and adolescents: a systematic review. Psychol Sport Exerc.

[9] Babic MJ, Morgan PJ, Plotnikoff RC, Lonsdale C, White RL, Lubans DR (2014). Physical activity and physical self-concept in youth: systematic review and meta-analysis. Sports Med.

[10] Barnett LM, Morgan PJ, Van Beurden E, Ball K, Lubans DR (2011). A reverse pathway? Actual and perceived skill proficiency and physical activity. Med Sci Sports Exerc.

[11] Ridgway CL, Ong KK, Tammelin TH, Sharp S, Ekelund U, Jarvelin M-R (2009). Infant motor development predicts sports participation at age 14 years: Northern Finland birth cohort of 1966. PLoS ONE.

[12] Barnett LM, van Beurden E, Morgan PJ, Brooks LO, Beard JR (2009). Childhood motor skill proficiency as a predictor of adolescent physical activity. J Adolesc Health.

[13] Lopes VP, Rodrigues LP, Maia JAR, Malina RM (2011). Motor coordination as predictor of physical activity in childhood. Scand J Med Sci Sports.

[14] Jaakkola T, Washington T (2012). The relationship between fundamental movement skills and self-reported physical activity during Finnish junior high school. Phys Educ Sport Peda.

[15] Iivonen S, Sääkslahti AK (2013). Preschool children’s fundamental motor skills: a review of significant determinants. Early Child Dev Care.

[16] Hesketh KD, Campbell K, Salmon J, McNaughton SA, McCallum Z, Cameron A, Ball K, Gold L, Andrianopoulos N, Crawford D (2013). The Melbourne Infant Feeding, Activity and Nutrition Trial (InFANT) Program follow-up. Contemp Clin Trials.

[17] Cain K, Sallis J, Conway T, Van Dyck D, Calhoon L (2013). Using accelerometers in youth physical activity studies: a review of methods. J Phys Act Health.

[18] Evenson K, Catellier D, Gill K, Ondrak K, McMurray R (2008). Calibration of two objective measures of physical activity for children. J Sports Sci.

[19] Trost SG, Fees BS, Haar SJ, Murray AD, Crowe LK (2012). Identification and validity of accelerometer cut-points for toddlers. Obesity.

[20] Trost SG, Loprinzi P, Moree R, Pfeiffer K (2011). Comparison of accelerometer cut points in predicting activity intensity in youth. Med Sci Sport Exerc.

[21] Ulrich DA (2000). Test of Gross Motor Development.

[22] Barnett LM, Minto C, Lander N, Hardy LL (2014). Interrater reliability assessment using the Test of Gross Motor Development-2. J Sci Med Sport.

[23] Harter S, Pike R (1984). The pictorial scale of perceived competence and acceptance for young children. Child Dev.

[24] Barnett LM, Ridgers ND, Zask A, Salmon J (2015). Face validity and reliability of a pictorial instrument for assessing fundamental movement skill perceived competence in young children. J Sci Med Sport.

[25] Barnett LM, Vazou S, Abbott G, Bowe SJ, Robinson LE, Ridgers ND, Salmon J (2016). Construct validity of the pictorial scale of Perceived Movement Skill Competence. Psychol Sport Exerc.

[26] Hnatiuk J, Salmon J, Campbell K, Ridgers N, Hesketh K (2013). Early childhood predictors of toddlers’ physical activity: longitudinal findings from the Melbourne InFANT Program. Int J Behav Nutr Phys Act.

[27] Cohen KE, Morgan PJ, Plotnikoff RC, Barnett LM, Lubans DR (2015). Improvements in fundamental movement skill competency mediate the effect of the SCORES intervention on physical activity and cardiorespiratory fitness in children. J Sports Sci.

[28] Logan SW, Robinson LE, Wilson AE, Lucas WA (2012). Getting the fundamentals of movement: a meta-analysis of the effectiveness of motor skill interventions in children. Child Care Health Dev.

[29] Barnett LM, Hinkley T, Okely AD, Salmon J (2013). Child, family and environmental correlates of children’s motor skill proficiency. J Sci Med Sport.

[30] Barnett LM, Lai SK, Veldman SLC, Hardy LL, Cliff DP, Morgan PJ, Zask A, Lubans DR, Shultz SP, Ridgers ND, et al. Correlates of gross motor competence in children and adolescents: a systematic review and meta-analysis. Sports Med. 2016:1–26. doi:10.1007/s40279-016-0495-z. 10.1007/s40279-016-0495-z PMC5055571 26894274

[31] Robinson LE, Wadsworth DD, Peoples CM (2012). Correlates of school-day physical activity in preschool students. Res Q Exerc Sport.

[32] Jones RA, Hinkley T, Okely AD, Salmon J (2013). Tracking physical activity and sedentary behavior in childhood: a systematic review. Am J Prev Med.

[33] Robinson LE (2010). The relationship between perceived physical competence and fundamental motor skills in preschool children. Child Care Health Dev.

[34] Barnett LM, Ridgers ND, Salmon J (2015). Associations between young children’s perceived and actual ball skill competence and physical activity. J Sci Med Sport.

[35] Liong G, Ridgers N, Barnett LM (2015). Associations between skill perceptions and young children’s actual fundamental movement skills. Percept Mot Skills.

[36] Slykerman S, Ridgers ND, Stevenson C, Barnett LM. How important is young children’s actual and perceived movement skill competence to their physical activity? J Sci Med Sport. 2016;19(6):488-92. doi:10.1016/j.jsams.2015.07.002. Epub 2015 Jul 10. 10.1016/j.jsams.2015.07.002 26232866

